# Conflicted between Goal-Directed and Habitual Control, an fMRI Investigation

**DOI:** 10.1523/ENEURO.0240-18.2018

**Published:** 2018-10-10

**Authors:** P. Watson, G. van Wingen, S. de Wit

**Affiliations:** 1 Department of Clinical Psychology, the Habit Lab, University of Amsterdam, Amsterdam 1018WT, The Netherlands; 2 Amsterdam Brain and Cognition, University of Amsterdam, Amsterdam 1018WT, The Netherlands; 3 Spinoza Centre for Neuroimaging, Academic Medical Center, University of Amsterdam, Amsterdam 1105AZ, The Netherlands; 4 Department of Psychiatry, Academic Medical Center, University of Amsterdam, Amsterdam 1105AZ, The Netherlands

**Keywords:** conflict, corticostriatal pathways, fMRI, goal-directed action, habit, slips-of-action task

## Abstract

“Slips of action” occur in everyday life when we momentarily lose sight of a goal (for example, when in a rush or distracted). Associative models propose that these habitual responses can be activated via a direct stimulus-response (S-R) mechanism, regardless of the current hedonic value of the outcome. The slips-of-action task (SOAT) has been extensively used in both healthy and pathological populations to measure habit tendencies, the likelihood of making erroneous responses for devalued outcomes. Inspection of behavioral performance does not reveal, however, whether the impairments were due to impaired goal-directed control or aberrantly strong habit formation. In the current study, we used functional MRI while human participants performed both the instrumental training and SOAT test phases, to elucidate the relative contributions of these mechanisms to performance on the SOAT. On trials in which conflict arises between competing goal-directed and habitual responses, we observed increased activation across areas including the anterior cingulate cortex, paracingulate gyrus, lateral orbitofrontal cortex (OFC), insula, and inferior frontal gyrus (IFG). Responding for devalued outcomes was related to increased activation in the premotor cortex and cerebellum, implicating these regions in habitual responding. Increased activation in the caudate, dorsolateral prefrontal cortex (dlPFC), and frontal pole during training was associated with better performance during the test phase, indicative of goal-directed action control. These results endorse interpretation of the SOAT in terms of competing goal-directed and habitual mechanisms and highlight that cognitive control processes present an additional bottleneck for successful performance on this task.

## Significance Statement

Imagine that you step in the car intending to drive to your new office but, distracted, end up driving the route to your old office instead. We are all familiar with the feeling that results from inadvertently carrying out a previously valid behavior, even if we no longer desire the consequence. In the current study, we examined how the brain reacts to cues that signal a previously rewarded response whose outcome value has now changed. We were also able to identify the brain regions that were activated when participants made an erroneous (habitual) response under time pressure. These results give us a richer sense of how the brain acts to control behavior when goal-directed processes are otherwise engaged.

## Introduction

Consider the following scenario: You intended to drop into the drycleaners on your way to work but in a hurry and momentarily distracted, you mindlessly follow your usual route, realizing your ‘slip of action’ halfway through the journey. This action slip is argued to reflect a stimulus-response (S-R) habit, triggered by the current context (e.g., the car) irrespective of one’s current goal. According to the associative-cybernetic model of instrumental action control, a S-R mechanism serves to activate behaviors that, on the basis of ones learning history, are likely to lead to a rewarding outcome. Furthermore, this mechanism can interact with a hedonic system that evaluates the outcome in light of one’s current needs and desires to produce goal-directed action that is based on the current goal status of the outcome (Dickinson, 1994, 2016; de Wit and Dickinson, 2009). However, under certain circumstances, the S-R mechanism can bypass the indirect goal-directed route altogether. For example, when one is rushing with little time to deliberate over the next course of action, the relatively fast, direct S-R mechanism may activate behavior before one has had the chance to evaluate the current hedonic value of the outcome. When outcome values change, this can lead to behavior that is not in line with current goals. The ability to monitor and resolve conflict by engaging inhibitory control may be crucial to avoid such action slips in everyday life.

The current fMRI study aims to reveal at the neural level the conflict between goal-directed and habitual processes that underlies habitual action slips. To this end, we adopted the “slips-of-action” test phase (slips-of-action task; SOAT), that has previously been applied in healthy participants (de Wit et al., 2012; Sjoerds et al., 2016; Snorrason et al., 2016) and has been used to provide evidence for reliance on habits in patient populations, including obsessive-compulsive disorder and addiction (Gillan et al., 2011; Delorme et al., 2016; Dietrich et al., 2016; Ersche et al., 2016). During the training phase, participants learn that discriminative cues signal which key presses yield valuable outcomes. During the test phase, some of these outcomes are now devalued through instruction (i.e., these now lead to deduction of financial credits). Participants are subsequently presented with a sequence of discriminative cues and must respond rapidly to cues that predict still-valuable outcomes while refraining from responding to stimuli that signal devalued outcomes. to reveal the competition between the fast S-R mechanism and the more indirect goal-directed pathway, the test phase is conducted under time pressure, such that participants have to rapidly decide whether to perform the learned response to each discriminative cue. Therefore, trials during which stimuli signal no-longer-valuable outcomes should reveal the competition between goal-directed and habitual processes, with time pressure tipping the balance toward habits (despite relatively brief instrumental training).

The present study is the first to investigate brain activations relating to action slips. The fMRI contrasts of main interest are between action slips (i.e., responses for devalued outcomes) and responses for valuable outcomes. We expected that these contrasts would reveal regions previously implicated in the balance between goal-directed and habitual control [respectively, ventromedial prefrontal cortex (vmPFC)/caudate and premotor cortex/posterior putamen; Valentin et al., 2007; de Wit et al., 2012; Morris et al., 2015; Delorme et al., 2016). Furthermore, because the speeded SOAT leads to conflict between the habitual and goal-directed pathways when outcomes are devalued, we expected that cognitive control processes present as much of a bottleneck for successful performance as the basic processes that underlie goal-directed action-outcome control and the formation and expression of S-R habits. Therefore, we additionally expect involvement of brain regions that have been implicated in the monitoring and resolution of response conflict, particularly as task difficulty increases (including anterior cingulate cortex (ACC) and paracingulate gyrus; Botvinick et al., 2004; Shenhav and Botvinick, 2015; Shenhav et al., 2016) and in the ability to inhibit prepotent responses [e.g., ACC and dorsolateral PFC (dlPFC); for review, see Verbruggen and Logan, 2008].

## Materials and Methods

### Participants

A total of 34 participants were tested. Of these, eight fMRI datasets were not usable due to issues with the scanner and/or the initial scanner protocol. Of the remaining 26 participants three were excluded from all analyses, one because he fell asleep in the scanner and two because they did not understand the task and responded for all outcomes (both valuable and devalued) during the test phase. The remaining 23 participants (five males) ranged in age from 18 to 30 (mean age: 21.9 years, SD: 3.0 years). The Psychology Ethics Committee of the University of Amsterdam approved the study.

### Stimuli and materials

#### SOAT

**Figure 1.**
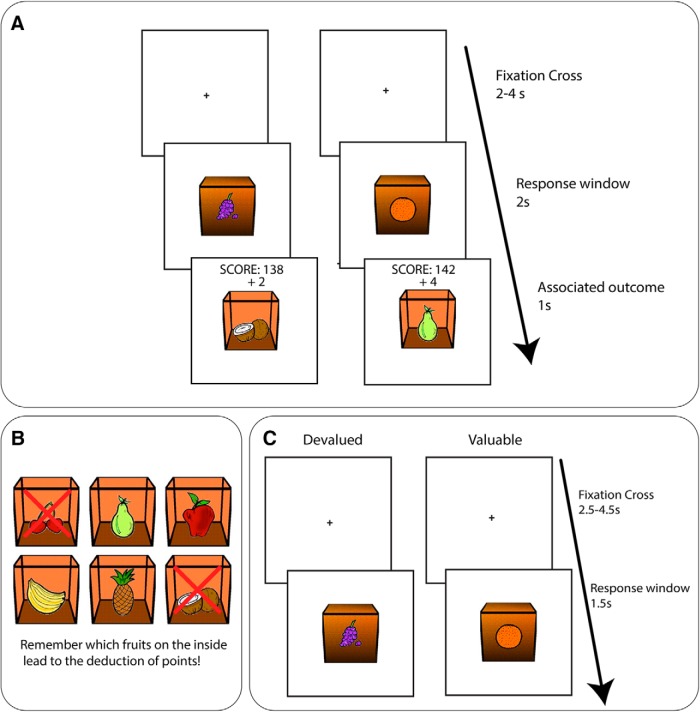
Task overview. ***A***, During the instrumental training phase participants saw fruits on the outside of the box and had to learn whether a left or right response was required to collect the fruit inside the box (and points). In this example, the orange stimulus is paired with the pear outcome, and the grape stimulus paired with the coconut. ***B***, Instructed outcome devaluation. At the beginning of each block, the six outcome fruits were shown and two were now devalued indicating that collecting them would lead to a deduction of points. ***C***, SOAT trials. Participants were shown the fruit stimuli in quick succession and had to respond when the associated fruit outcome was still valuable (e.g., pear is still valuable so a response should be made in the presence of the orange) or withhold responding if the fruit outcome was no longer valuable (e.g., the coconut is devalued so no response should be made to the grapes). No feedback was given although participants were told they were still winning (and losing) points.

Participants performed the instrumental learning phase and slips of action test phase of the “Fabulous Fruit Game” in the scanner. We used the same task version and stimuli as reported by Worbe et al. (2015) with any differences highlighted below. An overview of the task is depicted in Figure 1. In brief, during the instrumental training phase participants saw boxes with fruits on the outside and learned by trial and error the correct response (left or right) to make to gain different fruit outcomes inside the box (and points). Participants had 2 s in which to respond and the faster they responded the more points they earned. All feedback was presented for 1 s, displaying the total score and the points won on that trial. In addition, for correct responses the associated fruit outcome was shown during the feedback screen (inside the box; Fig. 1), for incorrect responses an empty box was displayed and “too late” presented on screen when no response was recorded. Participants completed 12 blocks of 12 trials in which a sequence of the six fruit pairs was randomly shuffled and shown twice (144 trials total). The ITI was 2–4 s selected at random, during which a fixation cross was presented.

During the slips-of-action test certain fruit outcomes were devalued, meaning that participants should no longer respond for those outcomes (as it would lead to the deduction of points) while continuing to respond for still valuable outcomes (and continue earning points for these). Each block began with the devaluation screen – for 5 s the six outcome pictures were shown and two of these were devalued as indicated by a red cross through them. Text underneath read “Remember which fruits on the inside lead to the deduction of points!” Participants then completed the fruitpicker test, they saw all six outcome pictures (arranged in a different order) and were asked to use the response keys to navigate around and select the two fruits which were devalued. If the incorrect fruits were selected the devaluation screen and fruitpicker test were repeated again. On each trial of the SOAT test phase, participants saw a fruit stimulus appear (for 1.5 s). During this 1.5-s response window, they had to make a decision whether to respond or not (depending on whether the stimulus predicted a valuable or a devalued fruit outcome). Participants did not receive feedback but were still earning one point for correctly responding (left or right) for each stimulus that predicted a still-valuable outcome but lost a point if they responded for a stimulus that predicted a now-devalued outcome. The ITI was 2.5–4.5 s, selected at random, during which a fixation cross was presented. Across nine blocks, all possible combinations of right-response and left-response paired outcomes were devalued. Each block consisted of 24 trials in which a random sequence of the six stimuli was shown four times (216 test trials in total). After blocks 3, 8, and 11 participants completed a filler block which was exactly the same as the test blocks except that there were no red crosses during the devaluation screen and participants were instructed that all the outcomes were now worth points. As such the filler blocks did not require participants to anticipate and evaluate the outcomes, they could instead rely on the S-R associations established during training. Filler blocks consisted of 12 trials in which the six stimuli were each shown twice (random order). In total therefore, participants completed 12 blocks. The total number of points (and corresponding financial reward) was then displayed on the screen.

### Procedure

Participants first completed a short demo of the training and test phases with eight different pictures of drinks (four functioned as stimuli and four as outcomes). This emphasized that faster responses would earn more points and that participants should try and learn the contingencies between stimuli, responses and outcomes. They were told that they should try and earn as many points as possible during the real task (and at the end all points would be converted into a financial bonus of up to €10). Participants were then taken to the scanner where they performed the SOAT and additional scans. After they had finished they returned to the lab room where they were tested on their knowledge of the S-R, R-O, and S-O contingencies. They then completed a demographic questionnaire and were paid €30 for their time plus their financial bonus earned during the task.

### Behavioral analyses

Given that the data were not normally distributed we used a Friedman test to examine RT and accuracy across the 12 blocks of the training phase. During the test phase response rates (%) on valued and devalued trials were compared with a Wilcoxon signed-rank test. The difference score [the devaluation sensitivity index (DSI), reflecting percentage of responses on valuable minus devalued trials] was used as an indication of performance in the subsequent MRI analyses. Accuracy on filler trials was calculated as an indication of participants retention of the S-R-(O) relationships.

### MRI data acquisition

Scanning was performed with a standard whole-head coil on a 3-T Siemens MRI system at the Spinoza Center (Academic Medical Center, Amsterdam). Participants viewed stimuli via a mirror and a projected image. Scanning consisted of two separate runs using a multi-echo sequence (three echoes; Poser et al., 2006) with 376 EPIs acquired during the training phase and 692 acquired during the SOAT test (SENSE acceleration factor R = 3.0, TR = 2.38 s; TEs = 9, 26.32, 43.63 ms; flip angle = 76°, 37 transverse slices, 3 × 3 × 3 mm + 10% interslice gap). To allow for equilibration of T1 saturation effects each run began with two dummy scans. After the functional runs, a 3D T1-weighted scan (TR = 8.3 ms; TE = 3.8 ms, flip angle = 8°, 220 slices, 1 × 1 × 1 mm, FOV = 240 × 188 × 220) was acquired.

#### fMRI preprocessing

A total of 30 extra scans from the end of the test phase were first used to combine the multi-echoes of each run into one volume (Poser et al., 2006). fMRI data analysis was then conducted with FSL FEAT (fMRI Expert Analysis Tool) version 6.0. In participant space, the fMRI time series were analyzed using an event-related approach in the context of the general linear model (Flame1+Flame2) with FILM prewhitening and standard motion correction. For the training phase, we collapsed the data across all correct trials. Two nuisance regressors modeled the incorrect trials and the instruction screens at the beginning and end. For the test phase, six separate regressors modeled (1) respond-valuable (trials in which the signaled outcome was valuable and the participant responded), (2) respond-devalued (slips-of-action trials in which the signaled outcome was devalued and the participant responded), (3) nonresponse-valuable (where the signaled outcome was valuable and the participant did not respond), (4) nonresponse-devalued (the signaled outcome was devalued and the participant did not respond), (5) respond-filler trials (all signaled outcomes were valuable and the participant responded correctly), and (6) a nuisance regressor modeling the instruction screens, fruitpicker trials, pauses, and incorrect filler trials leaving only the fixation cross period as the implicit baseline. All regressors were convolved with a double gamma HRF and its temporal derivative. The model was then high-pass-filtered (Gaussian-weighted least-squares straight-line fitting, with sigma = 50.0 s). Individual runs were visually inspected to ensure correct registration and the absence of excessive motion. The mean absolute displacement (each time point with respect to the reference image) was 0.04 mm (SD: 0.07 mm), mean displacement relative to the previous time point was 0.03 mm (SD = 0.04 mm). The relevant COPE images were registered to the high-resolution T1 image (using the BBR algorithm) and then non-linearly transformed to MNI standard space (using FNIRT, warp resolution 10 mm) before being finally merged into a single 4D file for statistical analyses.

#### fMRI higher-level analyses

Whole-brain analyses were performed on Z (Gaussianised T/F) statistic images. These were thresholded at *Z* = 3.1 and a corrected cluster threshold of *p* < 0.05 applied. to examine activation elicited by competition between goal-directed and habitual action control we first contrasted all devalued trials (both successful nonresponse and erroneous respond trials) > respond-valuable. Next, we followed this up by looking specifically at respond-devalued (i.e., slips of action) > respond-valuable trials in a subset of participants who made at least four slips during the test phase to account for behavioral responses in both conditions. We subsequently examined brain regions involved in goal-directed action control by contrasting respond-valuable > respond-devalued (slips) trials as well as respond-valuable>filler trials. To identify brain regions that mediate individual differences in the ability to perform in a goal-directed manner we used a series of single-group-average-with-covariate models, examining voxels whose mean activation on respond-valuable and nonresponse-devalued trials covaried with the behavioral DSI. Finally, we identified voxels whose mean activation during training blocks covaried with the DSI score, to determine in which regions activity during instrumental acquisition was a predictor of the ability to subsequently adapt behavior in a goal-directed manner. From all covariate analyses two participants were excluded as potential outliers (because of making an extreme number of slips of 40 and 47 relative to other participants). Note that whole-brain Z (Gaussianised T/F) statistic images (without thresholding) can be accessed at https://neurovault.org/collections/3989/.

## Results

### Behavior

#### Instrumental training phase

**Figure 2.**
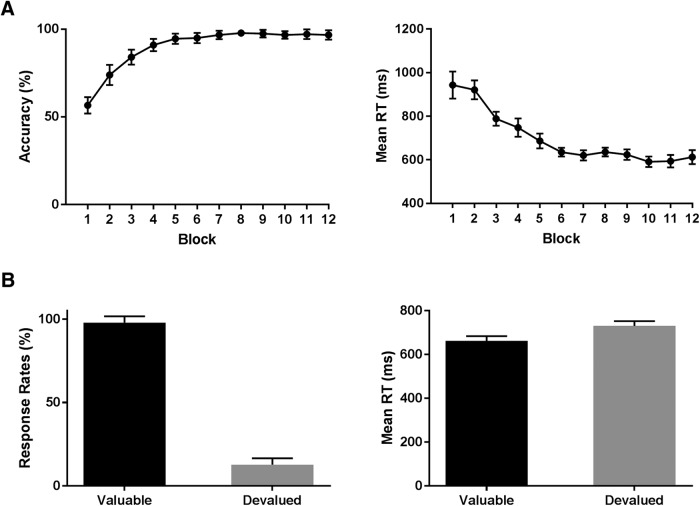
***A***, Mean accuracy and RT across the 12 blocks of training. Participants learned the correct response to make (left or right) in the presence of each stimulus to collect the outcomes. ***B***, Response rates and mean RT on the SOAT trials. In general, participants responded for valuable trials while withholding responses for devalued trials. The difference between valuable and devalued was calculated as the DSI. Responding for valuable outcomes was faster than responding for devalued outcomes during slips of action trials. Error bars represent standard error of the mean calculated using the Cousineau (2005) method.

As can be seen in Figure 2, participants learned by trial and error the correct response required in the presence of each of the six discriminative stimuli. As expected, participants became more accurate (and faster) over the course of training with a significant effect of block for both accuracy: χ*^2^*_(11)_ = 152.9, *p* < 0.001 and correct RTs: χ*^2^*_(11)_ = 142.0, *p* < 0.001. Median accuracy in the final block was 100% (IQR: 92–100%) with median RT on correct trials of 578 ms (IQR: 515–697 ms).

#### SOAT

During the slips-of-action test phase, participants had to respond for stimuli that signaled a still-valuable outcome while withholding responding for stimuli that signaled a devalued outcome. As expected, Wilcoxon signed-rank showed that participants responded significantly more on trials where stimuli predicted valuable outcomes relative to devalued (*Z =* 4.2, *p* < 0.001; Fig. 2). The median difference score (DSI) was 90% (IQR: 83–97%). The median number of slips trials was five trials (IQR: 2–11) with a total range of 1–47 slips trials from a possible total number of 72 devalued trials during the test phase. When examining mean RT, participants were significantly faster when responding for valuable outcomes as opposed to devalued outcomes, *t*_(22)_ = 3.06, *p* = 0.006.

#### Retention of contingency knowledge

During the test phase participants retained the knowledge of the S-R relationships as indicated by median accuracy of 97% (IQR: 97–100%) across the three filler blocks. When tested outside the scanner the median accuracy on the final tests of contingency knowledge was 100% (IQR: 100–100%).

### fMRI

#### Regions implicated in outcome devaluation

**Figure 3.**
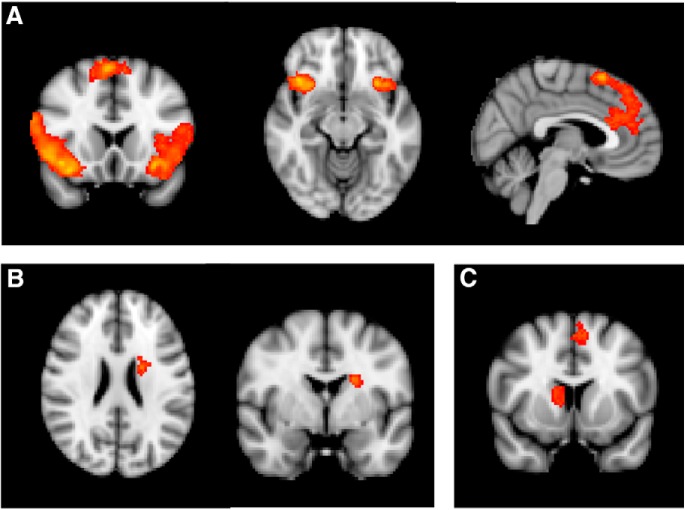
***A***, On devalued > respond-valuable trials, participants recruited regions associated with cognitive control and inhibition including ACC, paracingulate gyrus, and superior frontal gyrus in addition to bilateral OFC, insula, and IFG (at *x* = 4, *y* = 18, *z* = -12). ***B***, Caudate nucleus activation was associated with goal-directed control shown here for the respond-valuable > slips-of-action contrast (*y* = -2, *z* = 24). ***C***, Caudate nucleus and paracingulate gyrus were also activated on respond-valuable trials > filler trials (*y* = 10).

In contrast to valuable SOAT test trials, competition arises during devalued trials between the correct response signaled by the stimulus (S-R: respond) relative to the signaled outcome (O-R: do not respond), sometimes leading to a slip of action (an erroneous response). When contrasting mean activation on all devalued trials > respond-valuable trials (23 participants), activation was observed in a large cluster including the ACC, paracingulate gyrus and superior frontal gyrus extending into premotor cortex (peak voxel: 4, 18, 60; cluster size: 3575 voxels; Fig. 3) in addition to bilateral orbitofrontal cortex (OFC), inferior frontal gyrus (IFG), and insula activations (Fig. 3; Table 1). We repeated this analysis, restricting to devalued trials where a response was made (16 participants). This respond-devalued (slips of action) > respond-valuable contrast again revealed activation in the insula, lateral OFC, paracingulate gyrus and premotor cortex (for details, see Table 1).

**Table 1.** fMRI contrasts

Analysis	Cluster size	Cluster regions (Harvard Oxford atlas)	MAX *X* (mm)	MAX *Y* (mm)	MAX *Z* (mm)
All-devalued > respond-valuable	3575	Superior frontal gyrus	4	18	60
		Paracingulate Gyrus	-4	50	20
		ACC	8	36	22
	2462	Supramarginal gyrus	60	-44	28
	2392	Lateral OFC	44	24	-4
		IFG	50	22	2
		Insula	40	18	-10
	1272	Lateral OFC	-30	18	-12
		IFG	-40	22	2
		Insula	-30	18	-10
	690	Supramarginal gyrus	-58	-44	32
Slips-of-action trials > respond-valuable	1093	Insula	32	14	-14
		lateral OFC	42	22	-12
	368	Paracingulate gyrus	-4	50	20
	311	Insula	-34	16	4
		Frontal operculum cortex	-40	20	0
	283	Middle temporal gyrus	50	-34	-4
	215	Superior frontal gyrus (premotor cortex)	2	18	58
	204	Supramarginal gyrus	62	-44	30
Respond-valuable > slips-of action trials	148	Anterior caudate nucleus	-20	-2	24
	395	Occipital fusiform gyrus	24	-82	-2
Respond-valuable > filler trials	541	Superior parietal lobule	-6	-76	40
	279	Paracingulate gyrus	-6	16	48
	184	Anterior caudate nucleus	12	6	10

Exhaustive list of clusters after whole-brain correction for multiple comparisons at the cluster level (*p* < 0.05; cluster-forming threshold *Z* > 3.1).

#### Regions implicated in goal-directed control

**Figure 4.**
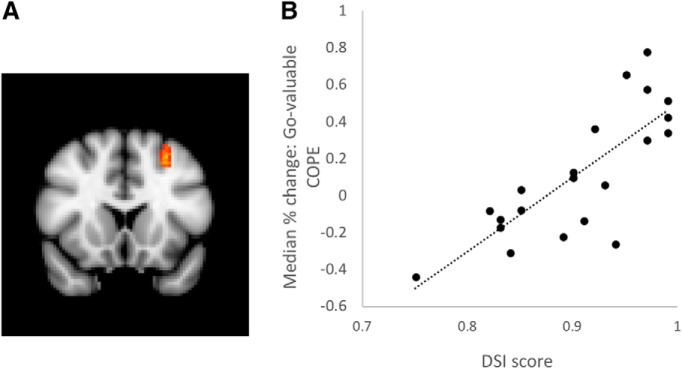
***A***, Increased activation in a region of dlPFC was associated with better performance on the SOAT test phase (*y* = 16 mm). ***B***, Scatterplot illustrating direction of relationship between activation on respond-valuable trials and DSI (higher score represents better goal-directed control). For illustrative purposes only. COPE = contrast of parameter estimate.

As expected, the respond-valuable > respond-devalued (slips-of-action) contrast revealed activation in the caudate nucleus (16 participants, Fig. 3: peak voxel: -20, -2, 24, cluster size: 148 voxels; see also Table 1), as well as in the occipital fusiform gyrus. We also contrasted respond-valuable trials in the SOAT test and filler blocks. During filler blocks all outcomes were valuable, meaning that there was no requirement to use O-R knowledge during these blocks. This contrast (23 participants) also revealed activation in caudate nucleus (peak voxel: 12, 6, 10, cluster size: 184 voxels) and paracingulate gyrus (peak voxel: -6, 16, 48; cluster size: 279 voxels; Fig. 3), implicating these regions in outcome retrieval and evaluation. Finally, during test trials on which participants responded for a valuable outcome, the covariance analysis identified a cluster in the dlPFC (21 participants, Fig. 4: peak voxel: -28, 16, 50, cluster size: 152 voxels) where mean activation covaried positively with DSI score. Similarly, with increasing DSI score, increased dlPFC activation was observed during trials on which participants successfully refrained from responding for a devalued outcome (-28, 14, 48, cluster size: 244 voxels). These analyses indicate that participants who engaged the dlPFC more were better able to exert goal-directed control.

#### Expression of goal-directed control is related to PFC activation during acquisition

**Figure 5.**
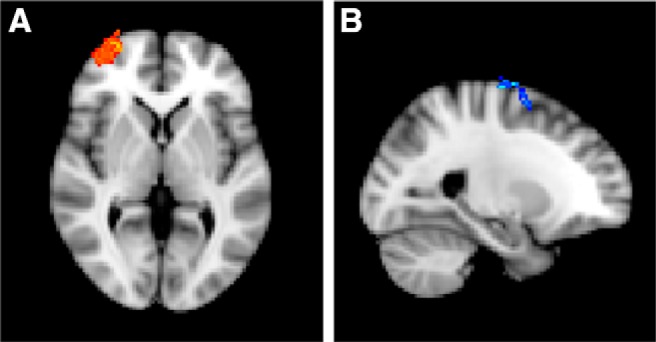
***A***, Increased activation in a region of frontal pole across the training phase corresponded to stronger goal-directed control during the test phase (*z* = 2 mm). ***B***, Increased activation in premotor cortex during training corresponded to stronger habit tendencies (more slips of action) during the test phase (*x* = -24 mm).

We correlated mean activation during the training phase with DSI score. The ability to resolve response conflict and act in a goal-directed manner on devalued trials (higher DSI score) correlated positively with activation during training in two clusters in the frontal pole (21 participants, peak voxel: 28, 62, 2; cluster size: 278 voxels; Fig. 5). Conversely, goal-directed performance during the test phase was negatively correlated with activation during training in the premotor cortex (21 participants, peak voxel: 10, -12, 78; cluster size: 364 voxels; Fig. 5) and cerebellum (Table 2), suggesting that these regions play a role in habit formation.

**Table 2.** Exhaustive list of clusters where mean activation across subjects covaried with DSI score (with higher scores indicative of better performance during the test phase)

DSI as additional covariate	Cluster size	MAX *X* (mm)	MAX *Y* (mm)	MAX *Z* (mm)	Cluster region
Mean respond-valuable activation (positive)	152	-28	16	50	Middle frontal gyrus (dlPFC)
Mean no-response-devalued activation (positive)	244	-28	14	48	Middle frontal gyrus (dlPFC)
Mean training activation (positive)	278	28	62	2	Frontal pole
	162	14	58	40	Frontal pole
Mean training activation (negative)	364	10	-12	78	Premotor cortex
	189	2	-76	-20	Cerebellum
	145	24	-36	-24	Cerebellum

Whole-brain analyses corrected for multiple comparisons at the cluster level (*p* < 0.05; cluster-forming threshold *Z* > 3.1).

## Discussion

The SOAT creates a situation in which goal-directed and habitual mechanisms activate conflicting responses (respond versus not respond), with time pressure favoring the faster habitual pathway. We observed activation on devalued trials (where such conflict arises) across areas including the ACC, paracingulate gyrus, lateral OFC, insula, and IFG, relative to respond-valuable trials. As expected, our results also implicate the premotor cortex and cerebellum in habitual control. Specifically, we found that premotor cortex activation was related to slips-of-action trials (respond-devalued relative to respond-valuable) and that increased activation in a more lateral region of premotor cortex during training in addition to the cerebellum was predictive of more slips of action during the test phase. In contrast, good performance during the test phase, indicative of goal-directed action control, was associated with increased activation in the caudate nucleus during respond-valuable trials (relative to both slips of action trials and the filler blocks), and with frontal pole activation during training. Finally, increased activation in a region of dlPFC during successful test trials (respond-valuable and no response-devalued) correlated with successful performance during the SOAT. Therefore, this study implicates the premotor cortex and cerebellum in habitual control and the caudate, frontal pole, and regions supporting broader cognitive control processes in goal-directed action, specifically in the face of conflicting S-R associations and under time pressure. These findings will be discussed in more detail below.

In everyday life we need to override habitual response tendencies (triggered by familiar contexts) to behave flexibly. We show that this competition between goal-directed and habitual action relates to activation of brain regions previously implicated in conflict monitoring, rule learning and response inhibition as observed in the stop signal task (Verbruggen and Logan, 2008), reversal learning (Hampshire et al., 2012), instrumental discrimination tasks (de Wit et al., 2009; de Wit et al., 2012; Sjoerds et al., 2013; Eryilmaz et al., 2017), and switch tasks (Walton et al., 2010). The activation observed in ACC is similar to previous studies using a similar instrumental discrimination task, where response competition arises between O-R and S-R processes (de Wit et al., 2009, 2012; Eryilmaz et al., 2017). Traditionally, fMRI activation in the (dorsal) ACC was attributed to this region’s role in top-down control processes, monitoring for response conflict and assigning cognitive resources as required when task difficulty increases (Milham et al., 2003; Botvinick et al., 2004; Shenhav and Botvinick, 2015; Shenhav et al., 2016). Recently however, alternative views argue that the ACC is more actively involved in decision-making - assessing possible courses of action afforded by the environment, integrating expected reward signals and directing action as required (Ebitz and Hayden, 2016; Kolling et al., 2016; Vassena et al., 2017). Both accounts fit with the ACC activation pattern found in the current study and we are unable to tease apart these possible contributions of the ACC to performance on the SOAT. We also found that lateral OFC, insula and IFG were activated more during the devalued trials. These regions are all important for conflict monitoring and likely represent the attempt to control prepotent responding elicited by the stimulus (Swick et al., 2008; Hampshire et al., 2010, 2012; Walton et al., 2010). It is noticeable that the paracingulate gyrus was seen to be active on both devalued trials and respond-valuable (relative to filler) trials. This rostral cingulate zone is strongly interconnected with dlPFC as well as ventromedial and frontopolar OFC and is argued to be activated in monitoring for unfavorable outcomes and increased cognitive control requirements (for review, see Ridderinkhof et al., 2004). A recent meta-analysis of different paradigms involving response inhibition reports that the paracingulate gyrus was consistently activated regardless of task (Zhang et al., 2017), reflecting the role of this region in the fronto-parietal network that supports adaptive control across diverse cognitive tasks (Cole et al., 2013). Our results suggest that during the SOAT test blocks participants were monitoring for devalued outcomes and potential conflict (regardless of trial type) but that this was not the case during the filler blocks.

In addition to these regions that support broader cognitive control processes we also found evidence of brain regions that are implicated more specifically in habitual versus goal-directed control. These findings are secondary, yet important, because they endorse behavioral studies that have used the SOAT to gain insight into the balance between habitual and goal-directed control in psychopathologies, interpreting poorer performance as evidence for increased habit propensity (Gillan et al., 2011; Ersche et al., 2016). Inspection of behavioral performance does not reveal whether the impairments were due to impaired goal-directed control or aberrantly strong S-R habit formation (Watson and de Wit, 2018), but the present findings suggest that fMRI can be used to elucidate the relative contributions of these mechanisms to performance on the SOAT. Importantly, we implicate different regions in habitual control (premotor cortex and cerebellum) versus goal-directed control (caudate, frontal pole, and dlPFC) and these results support the interpretation of performance on this task in terms of competing habitual and goal-directed pathways. Increased activation during training in motor regions was related to poorer performance (more slips-of-action) at test. Both cerebellum and premotor cortex activation likely reflects the development of behavioral automaticity during training as has been observed in the skill-learning literature (Doyon et al., 2003; Kelly and Garavan, 2005; Poldrack et al., 2005; Diedrichsen and Kornysheva, 2015). Cerebellum activation was specifically related to outcome devaluation insensitivity (Liljeholm et al., 2012), and structural MRI studies have previously related premotor cortex-striatal connectivity to increased slips-of-action (de Wit et al., 2012; Delorme et al., 2016). In contrast to this implementation of automaticity in the motor network for inflexible behavior, cortical regions such as the frontal pole and dlPFC (in addition to the anterior striatum) are involved in flexible action control that is sensitive to shifts in outcome value. The anterior caudate nucleus is commonly observed in instrumental reward learning and goal-directed behavior (O’Doherty et al., 2004; Liljeholm et al., 2012; Morris et al., 2015) and in addition to the dlPFC, is reported to be involved in the encoding of outcome representations at the time of responses (McNamee et al., 2015). Using a sequential decision-making task, transcranial direct stimulation over the dlPFC was seen to reduce reliance on a flexible “model-based” strategy (akin to goal-directed control; Smittenaar et al., 2013). The frontal pole (or anterior PFC) has been ascribed higher level cognitive functions such as counterfactual reasoning (Rushworth et al., 2011), evaluating possible future action-outcome relationships (Tsujimoto et al., 2011; Doll et al., 2015; Mansouri et al., 2015), and arbitrating between goal-directed and habitual control in the sequential decision-making task (Lee et al., 2014). Our results suggest that richer encoding of R-O schema during training was related to better SOAT test performance.

As mentioned previously, various neuroimaging studies have investigated habitual versus goal-directed action control, building on previous animal work in this field (de Wit and Dickinson, 2009; Balleine and O’Doherty, 2010). These studies have used an array of different tasks, determining the degree of “goal-directedness” by examining food choice after satiation (Valentin et al., 2007; Morris et al., 2015; Reber et al., 2017), manipulating the contingency between responses and outcomes (Tanaka et al., 2008), forcing participants to rely on S-R or R-O strategies during learning (de Wit et al., 2009; Liljeholm et al., 2012, 2015), or using a computational modeling framework to assess the degree to which participants use a flexible model-based strategy to maximize reward on subsequent trials (Daw et al., 2011; Lee et al., 2014). In general, these studies have sought to establish the neural correlates of value encoding that ultimately drives human choice behavior (Liljeholm and O’Doherty, 2012; Howard and Kahnt, 2017). The SOAT, by contrast, creates a situation where participants are triggered by external stimuli to respond for the devalued outcome and must attempt to override this slip of action. This is a scenario that occurs often in daily life but is difficult to establish in the lab (Watson and de Wit, 2018) and has not previously been studied using fMRI. The results sit well within the existing goal-directed/habit literature (as outlined above) but also offer novel insights into the role of control processes in managing this competition between goal-directed and habitual processes. It should be noted however that, unexpectedly, we did not find any evidence for the involvement of the putamen or the vmPFC in habitual and goal-directed control, respectively. The putamen has been consistently reported in functional and structure MRI studies using outcome devaluation and sequential-decision-making (model based/model free) paradigms (de Wit et al., 2012; Delorme et al., 2016; for meta-analysis, see Patterson and Knowlton, 2018). Likewise, vmPFC activation is often observed when contrasting O-R and S-R conditions (Valentin et al., 2007; McNamee et al., 2015; Eryilmaz et al., 2017). There are a number of possible explanations for why the current study did not find activation in these regions. In regard to the posterior putamen, one possibility is that the simple contrasts examining actions for devalued relative to still-valuable outcomes in the current study differ from previous approaches that have, for example, investigated S-R habits by modeling overall increases in task activation (relative to rest) after minimal and extensive behavioral repetition (Tricomi et al., 2009). Alternatively, there are some limitations to the current study which may have reduced the ability to detect activation in these aforementioned regions. Participants performed very well, making minimal slips of action, and analyses involving slips-of-action trials could only be conducted on a subset of participants. We made extensive modifications to the procedure of the current study, using a simpler version of the task than previously used (de Wit et al., 2012) and giving participants extensive instructions and demos beforehand (demonstrating the difficulty and speed of the test phase). While these modifications certainly reduced noise in the data, they may have had the unintended effect of ensuring that flexible goal-directed processes remained engaged throughout the task, leading to less slips-of-action trials and possibly less differential activations on respond-valuable > slips-of-action trials.

In summary, the current study investigated the response competition that arises when opposing responses are activated via the habitual S-R (respond) and goal-directed O-R (do not respond) associative chains. We observed that such conflict recruits brain regions associated with attentional control and inhibition including ACC, paracingulate gyrus, lateral OFC and IFG. Successfully overcoming this response conflict and acting in a goal-directed manner was associated with activation in the caudate nucleus and dlPFC.

## Synthesis

Reviewing Editor: Morgan Barense, University of Toronto Decisions are customarily a result of the Reviewing Editor and the peer reviewers coming together and discussing their recommendations until a consensus is reached. When revisions are invited, a fact-based synthesis statement explaining their decision and outlining what is needed to prepare a revision will be listed below. The following reviewer(s) agreed to reveal their identity: Peter Rudebeck, Matthew Rushworth. Note: If this manuscript was transferred from JNeurosci and a decision was made to accept the manuscript without peer review, a brief statement to this effect will instead be what is listed below.

We were all in agreement that appropriate revisions had been made following its previous submission to Journal of Neuroscience. We believe that it provides a clear and informative summary of the task that addresses an issue of interest to many researchers.
